# Inhibition of PARP Potentiates Immune Checkpoint Therapy through miR-513/PD-L1 Pathway in Hepatocellular Carcinoma

**DOI:** 10.1155/2022/6988923

**Published:** 2022-04-13

**Authors:** Guoqiang Sun, Ganggang Miao, Zhitao Li, Wubin Zheng, Chunguang Zhou, Guangshun Sun, Hongyong Cao, Zhouxiao Li, Weiwei Tang

**Affiliations:** ^1^Department of General Surgery, Nanjing First Hospital, Nanjing Medical University, Nanjing, Jiangsu, China; ^2^Department of General Surgery, Danyang People's Hospital, Danyang, Jiangsu, China; ^3^International Peace Maternity and Child Health Hospital, School of Medicine, Shanghai Jiao Tong University, Shanghai, China; ^4^Department of Plastic and Reconstructive Surgery, Shanghai Ninth People's Hospital, Shanghai Jiao Tong University School of Medicine, Shanghai, China; ^5^Hepatobiliary Center, The First Affiliated Hospital of Nanjing Medical University, Key Laboratory of Liver Transplantation, Chinese Academy of Medical Sciences, NHC Key Laboratory of Living Donor Liver Transplantation (Nanjing Medical University), Nanjing, Jiangsu, China

## Abstract

**Background:**

The DNA repair enzyme poly(ADP-ribose) polymerase (PARP) is involved in DNA damage repair and cell death. However, the association between PARP's biological activities and the immune microenvironment in hepatocellular carcinoma (HCC) is unclear. The present study will explore whether combining a PARP inhibitor with anti-PD1 might improve the anti-HCC impact and explain how it works.

**Method:**

The PARP inhibitor olaparib was screened out of 867 drugs through Cell Counting Kit 8 (CCK-8) assay. The expression of PARP was verified through the TCGA and TISIDB databases. The impacts exerted by PARP inhibitor olaparib to HCC cells were assessed via wound healing, Transwell, and proliferation assay. In vivo, experiments were performed in a C57BL/6 mouse model to evaluate the function of PARP inhibitor olaparib combination with anti-PD1 in HCC and mice tumors were further detected by immunohistochemically staining.

**Result:**

Olaparib was selected as the research object on the basis of drug screening. The results of the TCGA and Human Protein Atlas databases revealed that PARP was significantly upregulated in carcinoma cell cluster of HCC tissues compared to normal tissues. Higher expression of PARP showed a poorer prognosis based on Kaplan-Meier Plotter. qRT-PCR experiments confirmed that olaparib could increase PD-L1 expression through inhibiting miR-513 in HCC cells. In vivo, experiment confirmed that the combination of olaparib and anti-PD1 could enhance the immunotherapy effect of HCC.

**Conclusion:**

The present study reveals that inhibition of PARP potentiates immune checkpoint therapy through the miR-513/PD-L1 pathway in HCC and the combination of PARP inhibitor olaparib and anti-PD1 is beneficial to HCC therapy.

## 1. Introduction

Hepatocellular carcinoma (HCC) is the sixth most frequent cancer in the world and the third most deadly. There are more than 900,000 new HCC patients in the world every year, and nearly half of them are Chinese patients, of which 80% are primary liver cancer [1]. Specific to treatment of HCC include surgical resection, transcatheter arterial chemoembolization (TACE), and local ablation for early-stage disease [2]. However, the majority of HCC patients are diagnosed as advanced cancer due to HCC insidious onset. Therefore, the treatment of these patients is mainly chemotherapy and radiotherapy. In recent years, targeted therapy for HCC have made great progress and achieved encouraging results. Multitarget kinase inhibitors belong to a type of targeted therapy, including sorafenib, lenvatinib, regorafenib, and cabozantinib. Although these drugs prolong the survival period of HCC patients and improve the objective remission rate, such medicines increased median overall survival (OS) by less than 4 months and had a poor overall response rate in patients with advanced HCC [3]. Patients with HCC received a new treatment approach for immunotherapy with the introduction of immune checkpoint inhibitors. Currently, the US Food and Drug Administration (FDA) has approved four immune checkpoint inhibitors as single or combined therapy for the first-line/second-line treatment of HCC: atilizumab (programmed death-ligand 1 (PD-L1)) combined with bevacizumab (vascular endothelial growth factor (VEGF)), ipilimumab (cytotoxic T lymphocyte-associated antigen 4 (CTLA-4)) combined with nivolumab (programmed cell death protein 1 (PD1)), and single pembrolizumab (PD1) or single nivolumab. The total effective rate of anti-PD1 on HCC was only about 20%, indicating that anti-PD1 has little effect on most HCC patients [4, 5]. Therefore, a breakthrough in the treatment of HCC is needed to benefit more HCC patients.

PARP (poly(ADP-ribose) polymerase) is a kind of enzyme involved in a variety of cellular processes, including DNA damage repair. PARP inhibitors have been licensed for the treatment of breast and ovarian cancers caused by germline mutations in the BRCA1 and BRCA2 genes. PARP and BRCA genes are the key proteins involved in DNA repair in cells. PARP inhibitors prevent BRCA-mutated tumors from repairing DNA, causing them to collapse and cause cancer cells to die. Since there are fewer patients with HCC carrying BRCA mutant genes, PARP inhibitors have not been more extensively used in HCC [6].

PD-L1, also known as CD274, binds to PD1 receptor to block tumor-infiltrating lymphocytes. PD1 monoclonal antibody is the most widely utilized immunotherapy treatment for cancers in recent years. Nivolumab combined with ipilimumab received accelerated approval from the FDA in March 2020 for the second-line treatment of patients with advanced liver cancer who have received sorafenib treatment. The results of the Phase I/II clinical trial CheckMate-040 (NCT01658878) of nivolumab combined with ipilimumab as the second-line therapy for liver cancer showed that the overall response rate (ORR) reached 33%, twice that of other second-line therapies, and the sustained remission time was 4.6-30.5 months [7]. However, the primary endpoint overall survival (OS) in the CheckMate-459 phase III confirmatory clinical trial of nivolumab monotherapy did not approach statistical significance, and none of the primary endpoints OS and progression-free survival (PFS) were attained in the pembrolizumab confirmatory study KEYNOTE-240 [8]. Although there are still significant barriers to the application of immune checkpoint blockers in HCC, this indicates that the research field of HCC immunotherapy is broad.

Because fewer HCC patients have BRCA mutant genes [9], combining PARP inhibitors with other medications may be a viable option for improving the efficacy of PARP inhibitors on HCC. It reports that PARP inhibitors can cause immunological checkpoints such as PD1/PD-L1 to be upregulated in ovarian cancer [10]. The development of PD1/PD-L1 antibody combined with targeted drugs to treat HCC has become one of the focuses of many pharmaceutical enterprises in order to solve the heterogeneity and drug resistance of HCC. As a result, in the present study, we will investigate whether combining PARP inhibitor with PD1 monoclonal antibody might improve the anti-HCC impact and explain how it works.

## 2. Materials and Methods

### 2.1. Compound

867 small molecule compounds were purchased from Selleck (USA).

### 2.2. Cell Counting Kit 8 Assay

At a density of 4000 cells per well, HCC cells were implanted in 96 wells. Seed cells were treated with a 10 *μ*L Cell Counting Kit 8 (CCK-8) solution (RiboBio, China) for 0 h and 24 h of culture. The cell absorbance was measured at 450 nM at the appropriate time using a microplate analyzer, according to the manufacturer's instructions (Synergy4, USA).

### 2.3. Compound Screens

- Initial screening of 867 small molecule compounds: we obtained the proliferation rate of each compound on HCC cells through the Cell Counting Kit 8 experiment and judged the inhibitory effects of different drugs on liver cancer. The compound concentration is 10 *μ*M. This experiment was carried out in two cell lines, Hep-3b and YY-8103. For each drug, two cell lines were screened three times
- Second screening comparison: we used CCK-8 assay to compare the inhibitory effect of the compounds with the anti-HCC drugs sorafenib and sunitinib. The compound concentrations are 5 *μ*M and 10 *μ*M. This experiment was carried out in two cell lines, Hep-3b and YY-8103. For each drug, two cell lines were screened three times

### 2.4. Cell Cultures

Human HCC cell lines Hep-3b and YY-8103 were available from the Cell Bank of Type Culture Collection (Chinese Academy of Sciences, China). Hep-3b and YY-8103 cells were grown in Gibco's modified Eagle's medium (DMEM) (Gibco, USA) with 10% fetal bovine serum (FBS). The maintenance was conducted on overall cell lines under the temperature of 37°C within one constant-temperature incubator covering 5% CO_2_.

### 2.5. Quantitative Reverse Transcription Polymerase Reaction (qRT-PCR)

Total RNA from tissues and cells were isolated using a TRIzol reagent according to the producer's procedure (Invitrogen, USA). cDNA was synthesized for PD-L1 and miR-513 using a reverse transcription kit (Takara, Japan) and a RiboBio reverse transcription kit (RiboBio, China). The primers are human PD-L1, 5′-TCACTTGGTAATTCTGGGAGC-3′ (forward) and 5′-CTTTGAGTTTGTATCTTGGATGCC-3′ (reverse). GAPDH and U6 were used to standardize the expression levels of PD-L1 and miR-513 before the calculations.

### 2.6. Western Blot Analysis

HCC cells were treated with 10 *μ*M olaparib or dimethyl sulfoxide (DMSO) after 24 hours. Using a Mammalian Total Protein Extraction Kit and a protease inhibitor cocktail (Transgen, China), cellular proteins were extracted. Proteins were placed onto SDS-polyacrylamide gel and then transferred to polyvinylidene fluoride (PVDF) membranes (Wobixin Inc., China). Membranes were blocked with 5% skim milk for 1.5 hours after being rinsed with Tris-buffered saline and 1% Tween 20 (TBST, zsbb-bio, China). Then, at 4°C, we incubate with the primary antibody against: PD-L1 (ab213524, 1 : 1000 dilution) and GADPH (ab9485, 1 : 2500 dilution). The Western blot bands were detected using an enhanced chemiluminescence assay and a peroxidase-conjugated secondary antibody (CST, Sigma-Aldrich, USA).

### 2.7. Cell Proliferation Experiments

Control HCC cells and cells that had previously been treated with olaparib 5 and 10 *μ*mol/L for 48 hours were employed in the cell proliferation tests. In the clone formation experiment, cells were seeded onto 6-well plates at a density of 1000 cells per well, followed by cultured in DuIbecco's modified Eagle's medium (DMEM) with 10% FBS. After 10 days, the cells were fixed with methanol and then stained with GIMSA. Colonies eventually got the imaging and counting. Hep-3b and YY-8103 cells were given 5 and 10 *μ*mol/L olaparib and incubated at 37°C for CCK-8 testing method. Following that, CCK-8 solution (RiboBio,China) was introduced into each well and incubated for 2 hours. At 450 nm, the absorbance was measured at 0, 24, and 48 hours.

### 2.8. Transwell Migration and Invasion Assays

After 48 hours of treatment with 5 and 10 *μ*mol/L olaparib or DMSO, Hep-3b and YY-8103 cells were seeded in upper chambers with 200 *μ*L of serum-free DMEM, following the manufacturer's instructions. For invasion testing, the Transwell chamber (Corning, USA) was paved with Matrigel mix (BD Biosciences, USA), but no Matrigel mix was used for migration experiments. To function as an HCC cell chemoattractant, DMEM and 10% FBS were introduced into the bottom compartment. After the 24-hour incubation period was completed, the top chambers were fixed and then stained with crystal violet (Kaigen, China) for 15 minutes. The photograph and count processes were given to the cell lines in three fields for the visualizing technique.

### 2.9. Wound Healing Assay

When seeding on 6-well culture plates was achieved, Hep-3b and YY-8103 cells received 5 *μ*M and 10 *μ*M olaparib procedure and DMSO control. Artificial linear wounds were eliminated on the fused cell monolayer using a conventional 20 *μ*L pipette tip. The gradual removal was applied to free floating cells and detritus trapped inside the well bottom. The medium was given the introduction, and the plate was incubated at 37°C. The scratch gap width was measured using one inverted microscope and then photographed at 0, 24, and 48 hours.

### 2.10. PARP-2 Expression Level and Clinicopathological Analysis

The expression of PARP-2 in HCC tissues and normal tissues was investigated using TCGA portal. PARP-2 expression in various tissues was investigated using the Human Protein Atlas database. UALCAN was utilized to examine PARP-2 expression in HCC patients of various types, stages, grades, and nodal metastatic status in this study.

### 2.11. PARP-2 Expression Level and Survival Analysis

The Kaplan-Meier Plotter was used to analyze the relationships between PARP-2 expression and OS, progression-free survival (PFS), disease-specific survival (DSS), and recurrence-free survival (RFS). To compare the two groups of cases, the Kaplan-Meier survival plot was employed, and hazard rates with log rank *p* values and 95% confidence intervals were obtained.

### 2.12. Tools for PARP-2 Location in Cells

PARP-2 position in cells was determined using the Human Protein Atlas, which includes various studies on tissue, cell, and pathology atlas formats, as well as gene data in cells and tissues.

### 2.13. Tool for Immune-Related Analysis of PARP-2

TISIDB was used to investigate the spearman correlations between PARP-2 and immune-modulator expression. TISIDB is an online portal for tumor and immune system interactions that integrates a variety of heterogeneous data.

### 2.14. Mouse Model

The animal experiment was authorized by Nanjing Medical University's animal management committee, and all experiment protocols and animal care followed the university's institutional ethical guidelines for animal-related experimental activities. For HCC study, we conducted the random separation of 20 male C57BL/6 mice aged 5 weeks in PBS, olaparib, anti-PD1 (BP0273, bioxcell, USA), and olaparib+anti-PD1 groups (*n* = 5 for the respective group). 1 × 10^6^ H22 cells underwent the subcutaneous injection in the mouse inguen. Olaparib was dissolved in 4% of DMSO, 30% of PEG300, and 66% ddH_2_O. The mice were administrated 50 mg/kg every 3 day by intraperitoneal injection. Mouse anti-PD1 was injected intraperitoneally at 6.6 mg/kg on the eight day and once per three days thereafter. The tumor growth was measured every 4 days until mice were sacrificed. The tumor volume was calculated using the following formula: *a* × *b*^2^/2 (the largest and smallest tumor sizes, respectively, are shown by *a* and *b*).We killed all the mice in the experiment and collected tumor sizes and weighed tumors for further experimental tests.

### 2.15. Immunohistochemistry

The specific antibody shown and a biotin-coupled secondary antibody are used to stain each tissue sample, followed by incubation with the antibiotin-protein-biotin-peroxidase complex. The *H*-score approach was used to grade the samples, which combined values of immune response intensity and tumor cell staining percentage. Multiplying the percentage of target protein positive cells by the strength score yielded the final immunohistochemistry score.

### 2.16. Statistical Analysis

We used GraphPad Prism 8.0 (USA) to conduct the studies, and a *p* value of 0.05 was reported with statistical significance. We compared continuous data in the two groups using an individual *t*-experiment.

## 3. Results

### 3.1. Compound Library Screening Showed that PARP Inhibitor Olaparib Had Better Inhibitory Effect on HCC Cells

**Figure 1:**
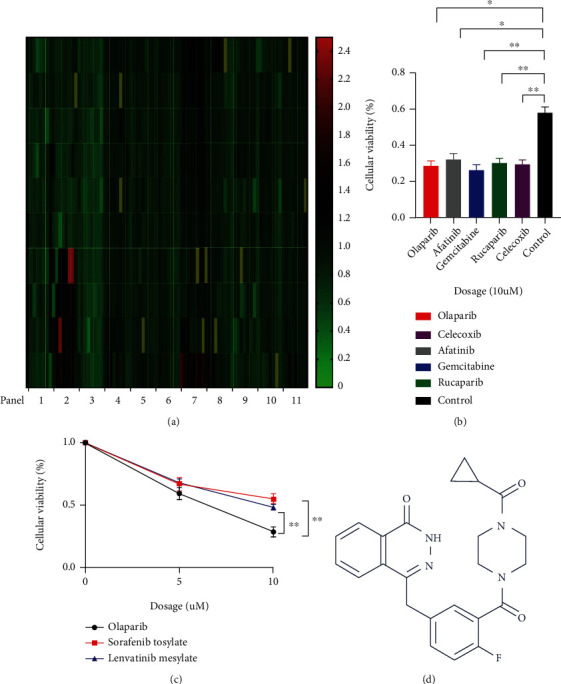
PARP inhibitor olaparib exerted prominent anti-HCC effect, screened from 867 compounds. (a) CCK-8 assays were employed in the first screening results: 867 compounds were separated into 11 panels, with various colors representing the ratio of HCC cell inhibition to the control group. (b) The five drugs with the best inhibitory effect on HCC cells using CCK-8 assays in the initial screening results: olaparib, afatinib, gemcitabine, rucaparib, and celecoxib. (c) Results of the rescreening used CCK-8 assays: comparison of the inhibition rates of olaparib and the drugs sorafenib tosylate and lenvatinib mesylate that have been marketed for the treatment of HCC at concentrations of 5 and 10 *μ*mol/L of HCC cells. (d) Olaparib compound structure (picture from http://www.selleck.cn) ^∗^*p* < 0.05, ^∗∗^*p* < 0.01.

Through the CCK-8 experiment, we conducted preliminary screening among 867 FDA-approved drugs in HCC cells. The results of screening were shown in Figure 1(a) and Supplementary Table 1. Analyzing the compounds that were effective in inhibiting HCC, we found that two (olaparib and rucaparib) of the top five most effective drugs belonged to PARP inhibitors (Figure 1(b)). Therefore, we believe that PARP inhibitors may have a greater killing effect on HCC cells, which greatly aroused our interest. Among the PARP inhibitors, we picked olaparib as our research subject. To better demonstrate the efficacy of olaparib in the treatment of HCC, we compared the small molecule inhibitors sorafenib, tosylate, and lenvatinib mesylate approved by the FDA for HCC as a comparison object with olaparib for HCC cell proliferation experiments. Experiment result showed that olaparib was better than the two inhibitors in inhibiting the proliferation of HCC cells (Figure 1(c)). The structure of olaparib is shown in Figure 1(d).

### 3.2. Olaparib Inhibited the Proliferation, Invasion, and Migration in HCC

**Figure 2:**
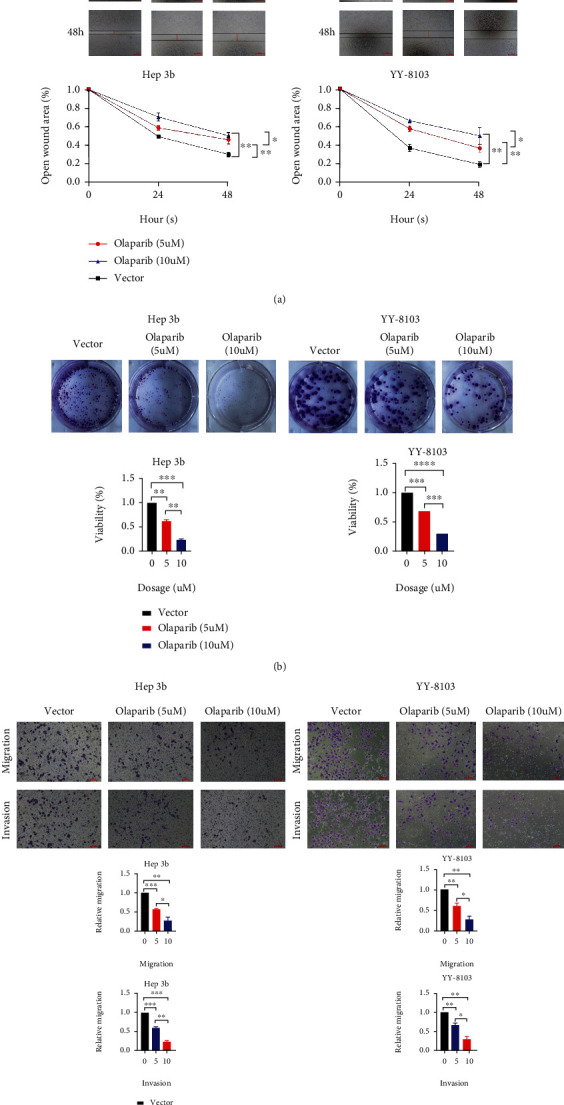
PARP inhibitor olaparib inhibited the proliferation, invasion, and migration in HCC. (a) Wound healing tests in HCC cells treated with 5 and 10 *μ*mol/L olaparib were used to investigate olaparib inhibition in HCC cells. (b) A clone formation experiment was utilized to assess the inhibitory effects of 5 and 10 *μ*mol/L olaparib. (c) After 5 and 10 *μ*mol/L olaparib culture, a Transwell experiment was utilized to measure cell invasion and migration. ^∗^*p* < 0.05, ^∗∗^*p* < 0.01, ^∗∗∗^*p* < 0.001, and ^∗∗∗∗^*p* < 0.0001.

In Hep-3b and YY-8103 cells, wound healing assay showed inhibition of olaparib to HCC cell migration, and 10 *μ*M olaparib exerted more powerful inhibition than 5 *μ*M olaparib (Figure 2(a)). Plate cloning experiments revealed that olaparib can decrease HCC cell proliferation, with 10 *μ*M showing a higher inhibitory effect than 5 *μ*M (Figure 2(b)). Transwell experiments were used to assess the effect of olaparib on HCC cell invasion and migration. Results revealed that olaparib inhibited the capacity of HCC cells to invade and migrate at various doses. At larger doses, olaparib has a stronger inhibitory ability, similar to wound healing assay and plate cloning experiments (Figure 2(c)). These results indicated that olaparib had a sufficient inhibitory impact on HCC cells.

### 3.3. Expression of PARP-2 in HCC Tissues

**Figure 3:**
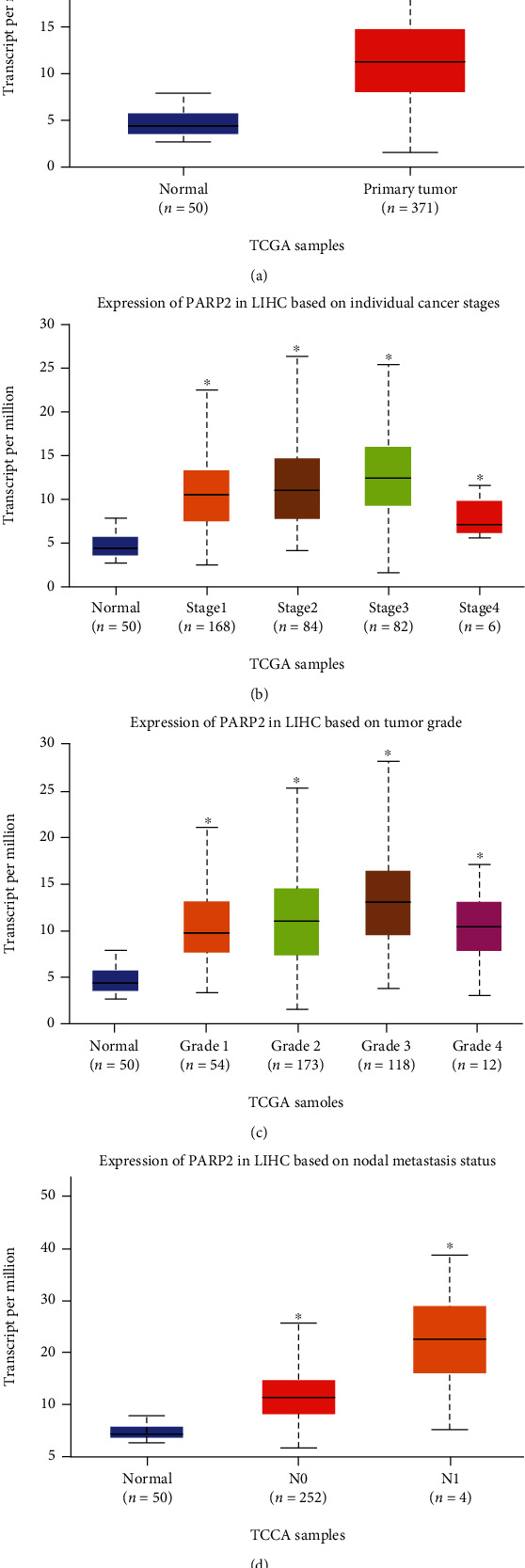
PARP-2 expression in HCC. (a) PARP-2 expression based on sample type. (b) PARP-2 expression based on individual cancer stages. (c) PARP-2 expression based on tumor grade. (d) PARP-2 expression based on nodal metastasis status. ^∗^*p* < 0.05.

**Figure 4:**
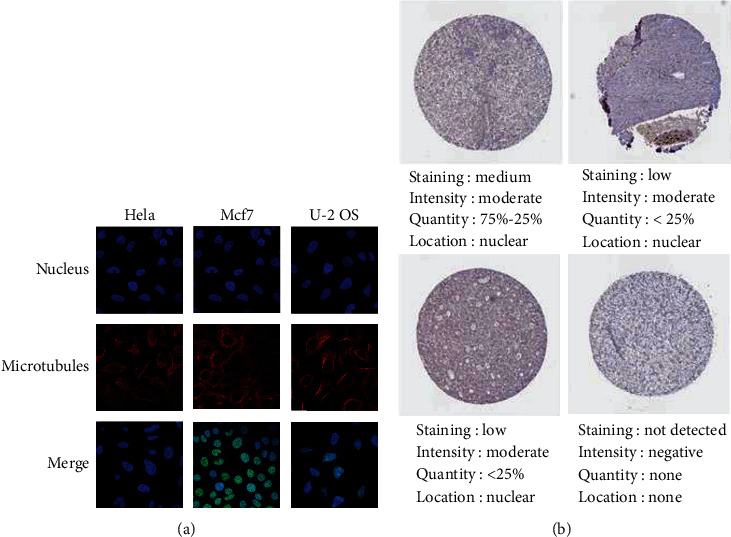
PARP-2 was expressed in HCC tissues. (a) PARP is found in a wide range of cancer cells. PARP-2 expression was found in the nucleus and cytoplasm of ovarian cancer Hela cells, breast cancer MCF-7 cells, and osteosarcoma U2-OS cells using immunofluorescence. (b) Expression of PARP-2 in tissues of multiple HCC patients by immunohistochemical staining.

The TCGA portal revealed that tumor tissues had higher PARP-2 expression than normal tissues (Figure 3(a)). The expression level of PARP-2 increased with the later stage of individual HCC samples, with stage 4 having a little lower expression level than the previous stages (Figure 3(b)). The relationship between PARP-2 expression and grade is similar to stage (Figure 3(c)). PARP-2 expression is much greater in individuals with HCC nodal metastasis than in normal individuals, and the degree of expression rises with nodal metastasis stage (Figure 3(d)). Furthermore, we analyzed the expression of PARP-2 in various malignancies using data from The Human Protein Atlas. Through immunofluorescence, PARP-2 was obviously expressed in the nucleus of human ovarian cancer Hela cells, human breast cancer MCF-7 cells, and human osteosarcoma U-2 OS cells, which indicated that PARP-2 expression is low cell line specificity (Figure 4(a)). According to data from The Human Protein Atlas, PARP-2 staining in HCC patient tissues is mostly moderate, and most HCC patient tissues express the protein (Figure 4(b)). Hence, all of these data pointed to PARP-2 being a promising target for HCC therapy.

### 3.4. Clinical Role of PARP-2 in HCC

**Figure 5:**
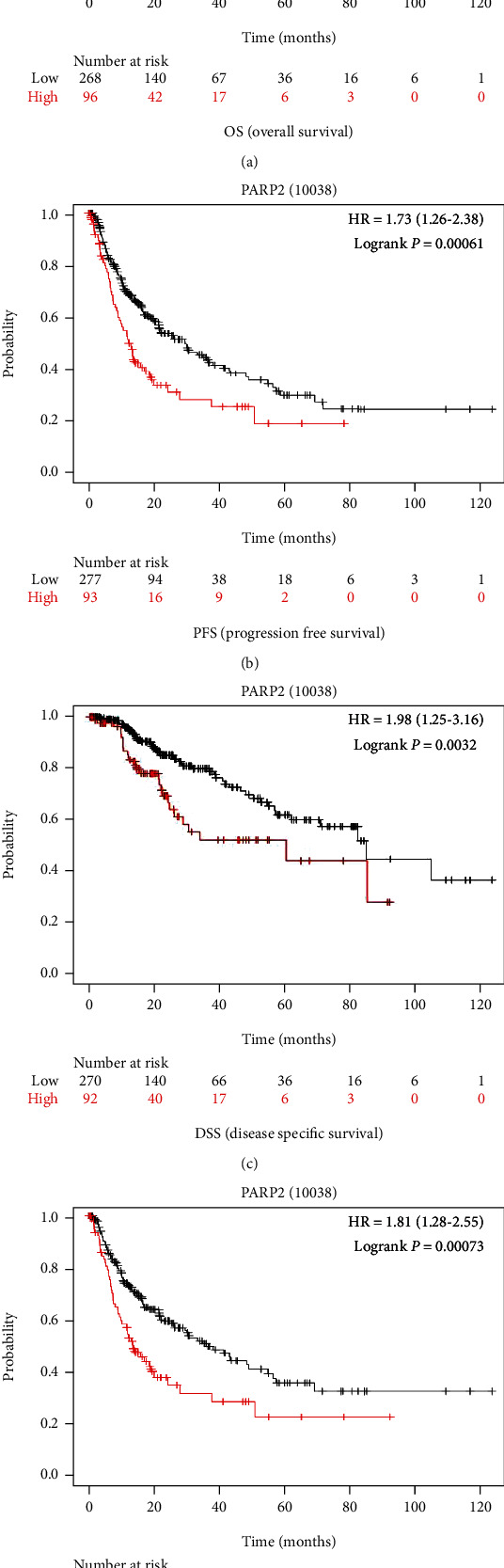
High expression of PARP-2 indicated a worse HCC clinical prognosis from Kaplan-Meier Plotter. (a) The association between PARP-2 and overall survival (OS) in HCC patients was shown by the Kaplan-Meier survival curve. *p* = 0.068 > 0.05. (b) In HCC patients, the Kaplan-Meier survival curve revealed an association between PARP-2 and progression-free survival (PFS). (c) In HCC patients, the Kaplan-Meier survival curve revealed an association between PARP-2 and disease-specific survival (DSS). (d) In HCC patients, the Kaplan-Meier survival curve revealed an association between PARP-2 and recurrence-free survival (RFS).

The Kaplan-Meier Plotter was used to further investigate the PARP-2 prognostic value in HCC. HCC patients with higher PARP-2 expression had poorer PFS, RFS, and DSS, according to the findings (Figures 5(b)–5(d)). HCC patients with increased PARP-2 expression had a lower OS, despite the fact that there was no significant difference (*p* = 0.068 > 0.05) (Figure 5(a)). As a result, individuals with HCC who have high PARP-2 expression might have a poor prognosis.

### 3.5. PARP-2 Expression Was Correlated with Immune Factors

**Figure 6:**
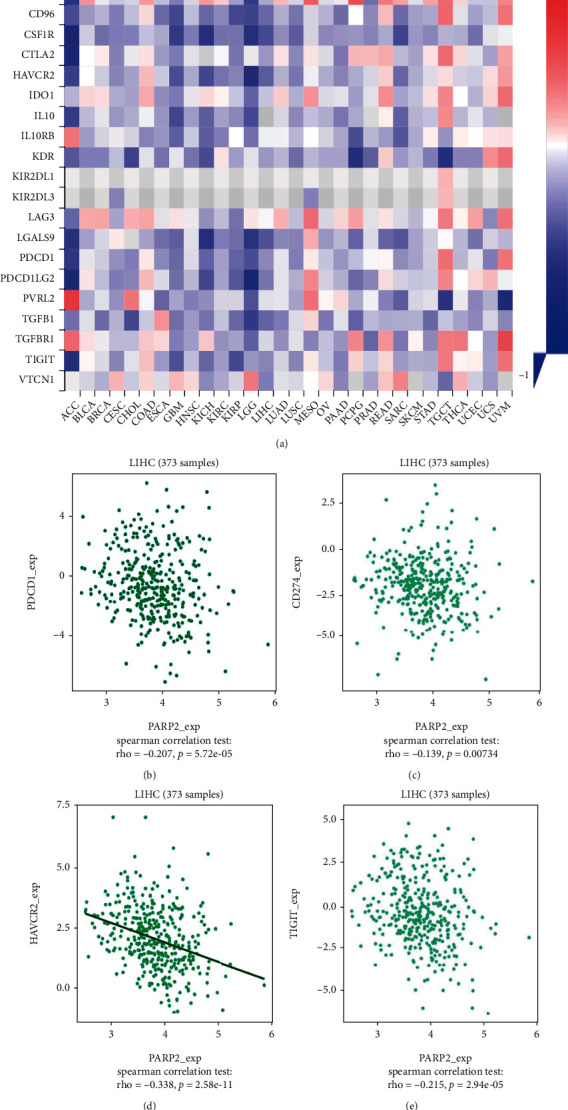
The relationship between PARP-2 and immunosuppressive factor. (a) Correlation between PARP-2 expression and various immunosuppressive factors. (b) The correlation between PDCD1 and PARP-2 in HCC. (c) The correlation between CD274 and PARP-2 in HCC. (d) The correlation between HAVCR2 and PARP-2 in HCC. (e) The correlation between TIGIT and PARP-2 in HCC.

The immune system had been previously reported related to the genesis and advancement of malignancies. As a result, we explored the connection between PARP-2 expression and immunological factors. In HCC, there was a substantial negative correlation between PARP-2 expression and immunosuppressive factors (CD274 (PD-L1), HAVCR2, IL10, etc.) as shown in Figure 6(a). The immunosuppressive factors PD1, CD274, HAVCR2, and TIGIT decreased when PARP-2 expression increased, as seen in Figures 6(b)–6(e). We selected PD-L1 as the target to further understand the impact of PARP-2 on HCC development, because the above results suggested that inactivation of PARP-2 expression might lead to the elevation of PD-L1 in HCC patients. There is a report that in ovarian cancer, PARP inhibitors can increase PD-L1 and affect the tumor immune microenvironment [10]. Hence, the combination of PARP-2 inhibition and immune checkpoint inhibitors might be useful in the treatment of HCC. We wondered that the mechanism of PARP-2 interact with immune checkpoints (e.g., PD-L1 and PD1).

### 3.6. The PARP Inhibitor Olaparib Caused Upregulation of PD-L1 by Inhibiting miR-513 in HCC Cells

**Figure 7:**
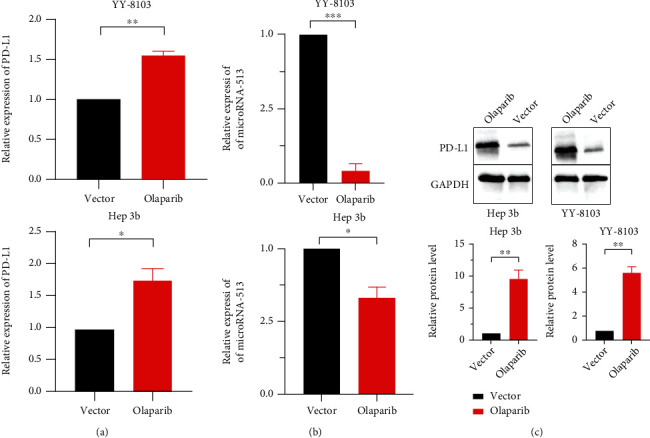
PARP inhibitor olaparib upregulated the expression of PD-L1 in HCC. (a) qRT-PCR detects the changes of PD-L1 caused by adding olaparib to HCC cells. (b) qRT-PCR detects the changes of miR-513 caused by adding olaparib to HCC cells. (c) In Hep-3b and YY-8102 cells, Western blot shows the effect of adding olaparib on PD-L1 protein expression. In comparison to the control group, the histogram also shows a rise in PD-L1 protein expression after adding olaparib 24 h. ^∗^*p* < 0.05, ^∗∗^*p* < 0.01, and ^∗∗∗^*p* < 0.001.

Using qRT-PCR to measure PD-L1 expression levels after olaparib was introduced to Hep-3b and YY-8103 HCC cell lines for 24 hours, we discovered that the expression level of PD-L1 in HCC cells increased, as predicted. After adding olaparib to both Hep-3b and YY-8103 cell lines, the expression of PD-L1 rose by around 1.5-fold (Figure 7(a)). The PD-L1 protein expression in the olaparib group was raised 24 hours after adding olaparib to HCC cells, according to the results of following Western blot tests, which were compatible with the qRT-PCR results (Figure 7(c)). We attempted to find the influence factors of the PD-L1 increase of HCC cells caused by olaparib. MicroRNAs (miRNAs) are 21-23 nt single-stranded regulatory RNA molecules, which regulate gene expression on the basis that they complement the 3′-untranslated region of the target mRNA and resulting in mRNA cleavage and/or translation inhibition. Gong et al. reported that miR-513 targets to PD-L1 3′-untranslated region and results in translational suppression [11]. qRT-PCR was used to detect miR-513 expression in HCC cells cultured with olaparib for 24 hours. Hep-3b cells and YY-8103 cells had lower expression of miR-513 compared with vector (Figure 7(b)). To sum up, PARP inhibitor olaparib induced increase of PD-L1 through repressing expression of miR-513 in HCC.

### 3.7. Inhibition of PARP Reduced HCC Tumor Progression and Enhanced the Efficiency of Anti-PD1 in HCC

**Figure 8:**
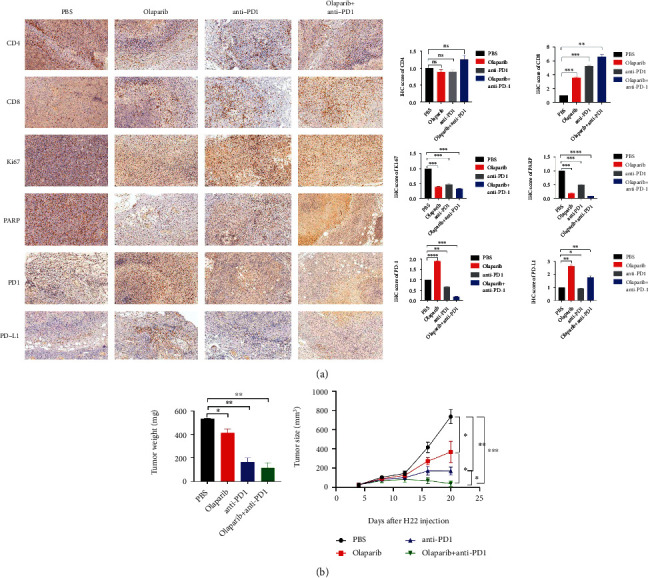
Inhibition of PARP-2 reduced HCC progression and enhanced the efficiency of PD1 monoclonal antibody in HCC. (a) Tumors were harvested after mice were sacrificed on day 20. Immunohistochemical staining of mouse tumors was used to detect the expression of CD4, CD8, Ki67, PARP, PD1, and PD-L1 in the PBS group, olaparib single-use group, anti-PD1 single-use group, and Oolaparib+anti-PD1 combination group. (b) The weight of tumors in each group after the mice were sacrificed. (c) The volume growth curve of mouse tumors. ^∗^*p* < 0.05, ^∗∗^*p* < 0.01, ^∗∗∗^*p* < 0.001, and ^∗∗∗∗^*p* < 0.0001.

**Figure 9:**
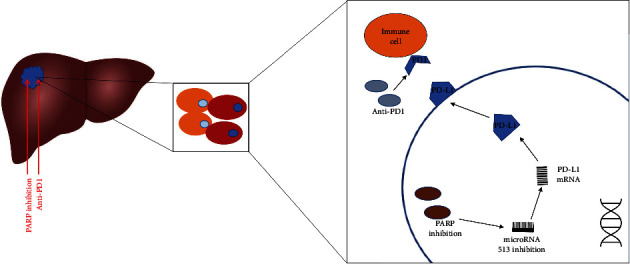
The mode pattern of PARP inhibitor olaparib in combination with anti-PD1 to improve HCC treatment. Inhibition of PARP potentiates immune checkpoint therapy through the miR-513/PD-L1 pathway in HCC and the combination of PARP inhibitor olaparib and anti-PD1 is beneficial to HCC therapy.

We injected H22 cells into the subcutaneous groins of C57BL/6 mice and then administered intraperitoneally olaparib or PD1 antibody alone or in combination to mice and analyzed tumor growth in 20 days, as well as tumor weight after the mice were killed. As indicated from the results, the olaparib group inhibited HCC growth compared with the control group, and the anti-PD1 group significantly inhibited HCC development in which HCC progression was almost halted after day 16 in particular. What is more surprising is that the combination group of olaparib combined with anti-PD1 had a better inhibitory effect on tumors, compared with the single-drug group and the PBS control group. After day 16, the volume of HCC tumor decreased (Figure 8(c)). The mice were killed on day 20, and tumor tissues were obtained and weighed. The tumor weights of the olaparib single-drug group and anti-PD1 single-drug group were both lighter than those of the control group, while the combination group of olaparib and anti-PD1 showed more excellent tumor volume reduction effect, consistent with tumor growth volume results(Figure 8(b)). Next, we took the HCC tumors for immunohistochemical analysis. Whether it was used as a single drug of olaparib and anti-PD1, or a combination of two drugs, the results of Ki-67 showed a reduced effect. As expected, the expression of PARP had a negative relationship with PD-L1 and PD1 expression. After using olaparib to inhibit the expression of PARP, the PD-L1 of the olaparib single-use group increased, and the PD-L1 and PD1 of the combination group of olaparib and anti-PD1 decreased. As for the results of immune factors, the changes of CD4 were not statistically different among the groups. The olaparib single-drug group, anti-PD1 single-drug group, and combination group of olaparib and anti-PD1 all showed upregulation of CD8 expression, and CD8 expression of the combination group increased more significantly, which proves that the PARP inhibitor olaparib combined with anti-PD1 can effectively enhance the effect of immunotherapy (Figure 8(a)). The present study reveals that inhibition of PARP potentiates immune checkpoint therapy through the miR-513/PD-L1 pathway in HCC and the combination of PARP inhibitor olaparib and anti-PD1 is beneficial to HCC therapy (Figure 9).

## 4. Discussion

In breast and ovarian tumors with BRCA gene mutations, PARP inhibitors were effective [12, 13]. In recent years, researchers have focused on the use of PARP inhibitors in pancreatic cancer. In patients with germline BRCA mutations and metastatic pancreatic cancer, a phase III clinical study found that the olaparib maintenance group had a longer median progression-free survival than the placebo group (7.4 months vs. 3.8 months) [14]. From our screening of 867 drugs for the inhibition of HCC, we found that PARP inhibitors have a definite inhibitory effect on HCC cells. The impact of the PARP inhibitor olaparib on HCC was then confirmed in vitro. According to data from databases (TCGA (The Human Protein Atlas)), PARP-2 is significantly correlated with HCC staging, tumor grade, and metastasis, and the high expression of PARP-2 presents worse prognostic indicators such as OS and PFS. Through the analysis of tumor tissues in the database, PARP-2 was found in a range of cancer tissues, including those from HCC patients. As a result, we concluded that the inhibition of PARP might be beneficial to patients with HCC.

PARP inhibitors, as we all know, have been authorized by the FDA for cancer patients with BRCA gene abnormalities. However, a data based on gene sequencing of 357 patients with primary liver cancer showed that only 4.8% of them carried the BRCA gene [9]. This indicates that patients with HCC are less likely to carry BRCA mutant genes, which makes the application of PARP inhibitors in HCC a certain obstacle. Researchers discovered that PARP inhibitors in ovarian cancer and breast cancer can cause PD-L1 overexpression, which is a biomarker for the success of anti-PD1 or anti-PD-L1 combo treatment [10, 15]. And in the TISIDB database, we also found that PARP2 is negatively correlated with the immunosuppressive factor PD-L1, so we hypothesize that PARP inhibitors combined with PD1 monoclonal antibody can enhance the effect of treating HCC. We have detected through qRT-PCR that olaparib can increase the PD-L1 of HCC cells, and Western blot corroborated the qRT-PCR results. To find out why PARP inhibitors cause an increase in PD-L1 in HCC cells, we confirmed that olaparib promotes the transcription of PD-L1 by inhibiting miR-513. These results clarify the specific mechanism for the treatment of HCC with olaparib combined with PD1 monoclonal antibody.

The trials of vivo indicated that olaparib had obvious anticancer effects. Olaparib alone had a significant inhibitory effect on HCC, according to immunohistochemically staining of mouse tumors. When compared to the PBS group, Ki-67 and PARP expressions were much lower, and tumor weight and volume were also significantly lower. Interestingly, the expression of PD-L1 was dramatically elevated in the olaparib single-use group, which is consistent with previous in vitro data and offers a factual foundation for the combination of olaparib and PD1 monoclonal antibody. The staining results of the combination group of olaparib and PD1 monoclonal antibody showed a stronger inhibitory effect on liver cancer. The tumor growth volume curve showed a significant decrease compared with the single-drug groups and the PBS group, and the final tumor weight was also significantly reduced. The immunohistochemically staining of the combination group showed that Ki-67 and PD1 had lower expression, the significant upregulation of CD8, which represents the effect of killing tumor cells, compared with other groups. In conclusion, our experiments had proved that olaparib could enhance the immunotherapy of HCC, which provided another explanation for the application of PARP inhibitors in HCC.

Our research still had limitations in some aspects. We had not already examined the mechanism by which PARP inhibitors boost PD-L1 expression in detail; therefore, additional study was needed. Moreover, while the drug had a therapeutic effect, it also had some side effects on the body. We had not explored the side effects of the combination of PARP inhibitors and PD1 monoclonal antibody. However, we believe that the combination of PARP inhibitors and PD1 monoclonal antibody is bound to bring prospect for treatment of more HCC patients.

## Data Availability

The datasets obtained and analyzed during the current study were available from the corresponding authors in a reasonable request.

## Supplementary Materials

Supplementary Materials Ratios of 867 compounds to controls are presented in Supplementary Table 1. This is cited in section of compound library screening result as supplementary Table 1.
