# Supplementary material for: Inhibition of PARP Potentiates Immune Checkpoint Therapy through miR-513/PD-L1 Pathway in Hepatocellular Carcinoma
Source: J Oncol. 2022 Apr 13;2022:6988923. doi: 10.1155/2022/6988923 (PMC9020948; doi:10.1155/2022/6988923)
Supplement: Supplementary Materials — Ratios of 867 compounds to controls are presented in Supplementary Table 1. This is cited in section of compound library screening result as supplementary Table 1. [file 6988923.f1.pdf]

**Supplementary Table 1. Ratio of 867 compounds to the control group**

| | Ratio of 867 compounds to the control group | | | | | | | | | |
|---|---|---|---|---|---|---|---|---|---|---|
| Panael 1 | Axitinib | Gefitinib (ZD1839) | Sorafenib Tosylate | Crizotinib | Docetaxel | Anastrozole | Cladribine | Methotrexate | Letrozole | Entecavir Hydrate |
| | 0.928221 | 0.91417 | 0.878449 | 0.915185 | 0.877941 | 1.261216 | 1.028949 | 1.195531 | 1.32453 | 1.117318 |
| | Roxadustat (FG-4592) | Imatinib Mesylate (STI571) | Sunitinib Malate | Vismodegib (GDC-0449) | Paclitaxel | Aprepitant | Decitabine | Bendamustine HCl | Temozolomide | Nepafenac |
| | 0.948874 | 0.76621 | 0.778229 | 0.817505 | 0.780599 | 0.852548 | 0.756899 | 0.94261 | 0.834264 | 0.814627 |
| | Nintedanib (BIBF 1120) | Lapatinib (GW-572016) Ditosylate | Temsirolimus (CCI-779, NSC 683864) | Belinostat (PXD101) | Capecitabine | Bicalutamide | Dutasteride | Epirubicin HCl | Tamoxifen | Rufinamide |
| | 0.774166 | 0.943795 | 0.776875 | 0.682411 | 0.836127 | 0.815135 | 0.88539 | 0.566108 | 0.864906 | 0.765194 |
| | Afatinib (BIBW2992) | Lenalidomide (CC-5013) | Vorinostat (SAHA, MK0683) | Rucaparib (AG-014699,PF-01367338) phosphate | Lenvatinib (E7080) | Fulvestrant | Melatonin | Etoposide | Vincristine sulfate | Posaconazole |
| | 0.492805 | 0.93787 | 0.742678 | 0.472073 | 0.77569 | 0.893855 | 0.764855 | 0.806331 | 0.727781 | 0.711359 |
| | Bortezomib (PS-341) | Panobinostat (LBH589) | Entinostat (MS-275) | Cabozantinib (XL184, BMS-907351) | Valproic acid sodium salt (Sodium valproate) | Raltitrexed | Bisoprolol fumarate | Raloxifene HCl | Agomelatine | Prasugrel |
| | 0.630608 | 0.745218 | 0.856949 | 0.872016 | 0.864737 | 0.895717 | 0.814965 | 0.996614 | 0.868969 | 0.761131 |
| | Bosutinib (SKI-606) | Nilotinib (AMN-107) | Enzastaurin (LY317615) | Everolimus (RAD001) | Regorafenib (BAY 73-4506) | Thalidomide | Tivozanib (AV-951) | Fludarabine Phosphate | Leflunomide | Ramelteon |
| | 0.695785 | 0.95463 | 0.767733 | 0.836973 | 0.715592 | 0.826985 | 0.724225 | 0.839343 | 0.845945 | 0.725241 |
| | Dasatinib | Pazopanib HCl (GW786034 HCl) | Olaparib (AZD2281, Ku-0059436) | Malotilate | Danoprevir (ITMN-191) | Exemestane | Doxorubicin (Adriamycin) HCl | Topotecan HCl | Enzalutamide (MDV3100) | Cinacalcet HCl |
| | 0.780599 | 0.888945 | 0.483832 | 0.868461 | 0.899949 | 0.821398 | 0.607754 | 0.783139 | 0.75893 | 0.842729 |
| | Ridaforolimus (Deforolimus, MK-8669) | Rapamycin (Sirolimus) | Masitinib (AB1010) | Ivacaftor (VX-770) | Ritonavir | Finasteride | Fluorouracil (5-Fluoracil, 5-FU) | 2-Methoxyestradiol (2-MeOE2) | Dienogest | Celecoxib |
| | 0.957847 | 0.933638 | 0.840697 | 1.151515 | 1.077535 | 1.201625 | 1.24344 | 1.141696 | 1.149822 | 0.466886 |
| Panel 2 | Ketorolac | Enalaprilat Dihydrate | Aminoglutethimide | Ipratropium Bromide | Hydrocortisone | Deferasirox | Azathioprine | Meloxicam | Nevirapine | Pitavastatin Calcium |
| | 0.908711 | 1.006001 | 0.962175 | 1.127114 | 0.955628 | 0.806874 | 1.068012 | 0.486253 | 1.059465 | 1.000727 |
| | Adenosine | Dofetilide | Aminophylline | Sulfanilamide | Desonide | Piroxicam | Indomethacin | Mesna | NEXIUM (esomeprazole magnesium) | Rifapentine |
| | 0.802328 | 0.811057 | 0.827605 | 0.737043 | 0.794508 | 0.69158 | 0.499568 | 1.255865 | 1.300055 | 1.15621 |

| | | | | | | | | | | |
|---|---|---|---|---|---|---|---|---|---|---|
| | Zolmitriptan | Isradipine | Lubiprostone | Betamethasone Dipropionate | Didanosine | Gemcitabine | Terbinafine | Methocarbamol | Nicotinic Acid | Suprofen |
| | 0.814512 | 0.874341 | 0.832515 | 0.710857 | 0.768322 | 0.418258 | 1.493181 | 1.429533 | 2.281688 | 1.61102 |
| | Telbivudine | Estrone | Amorolfine HCl | Meprednisone | Divalproex Sodium | Glipizide | Levonorgestrel | Prednisolone | Nimodipine | Pyrazinamide |
| | 0.851791 | 0.882706 | 0.767049 | 0.798873 | 0.736134 | 0.68267 | 1.365703 | 1.227678 | 1.185488 | 1.243681 |
| | Monobenzone | Flucytosine | Chloramphenicol | Betamethasone Valerate | Emtricitabine | Glyburide (Glibenclamide) | Gemfibrozil | Telmisartan | Nisoldipine | Quetiapine Fumarate |
| | 0.927805 | 0.905619 | 0.814875 | 0.774504 | 0.76323 | 0.945263 | 1.425168 | 1.231133 | 1.116021 | 1.172941 |
| | Tretinoin | Trichlormethiazide | Flurbiprofen | Praziquantel | Progesterone | Fomepizole | Indapamide | Thiabendazole | Octocrylene | Rifampin |
| | 0.908165 | 0.897436 | 0.783597 | 0.851246 | 0.745045 | 0.751227 | 2.321877 | 1.233315 | 1.250227 | 1.207856 |
| | Phenylbutazone | Loteprednol etabonate | Disulfiram | Busulfan | Lamivudine | Adefovir Dipivoxil | Mitotane | Guaifenesin | Oxybutynin | Cefditoren Pivoxil |
| | 0.994363 | 0.90762 | 0.834879 | 1.082379 | 0.692126 | 0.775777 | 2.249136 | 1.583379 | 1.253682 | 1.262957 |
| | Ezetimibe | | Mesalamine | Carbamazepine | Estradiol | Zalcitabine | Methylprednisolone | Rifabutin | Enoxacin | Sulfadiazine |
| | 1.036552 | | 0.981997 | 0.871249 | 0.752864 | 0.858156 | 0.94108 | 1.013275 | 0.989271 | 0.951082 |
| Panel 3 | Clindamycin palmitate HCl | Buflomedil HCl | Clinofibrate | Canagliflozin | Dabrafenib | Alogliptin benzoate | Icotinib | Amoxicillin | Fenoprofen calcium hydrate | Cinepazide maleate |
| | 0.678124 | 0.915072 | 0.864517 | 0.832152 | 0.928183 | 0.806993 | 0.755138 | 0.758209 | 0.714978 | 0.97992 |
| | L-Thyroxine | Fluocinonide | Ciprofibrate | Alectinib (CH5424802) | MPEP | Camostat Mesilate | Carbazochrome sodium sulfonate (AC-17) | Aspirin | Linagliptin | Otilonium Bromide |
| | 0.875738 | 0.79459 | 0.783605 | 0.79022 | 0.695252 | 0.814552 | 0.854477 | 0.890149 | 0.865344 | 0.816206 |
| | Gliclazide | Inulin | Dolutegravir (GSK1349572) | MK-2048 | Alpelisib (BYL719) | Prucalopride | Clevudine | Niflumic acid | Vildagliptin (LAF-237) | Bosentan Hydrate |
| | 0.812662 | 0.863336 | 0.824238 | 0.707182 | 0.622017 | 0.698086 | 0.749114 | 0.748405 | 0.682022 | 0.789511 |
| | Acemetacin | Lonidamine | Trametinib (GSK1120212) | Laquinimod | Clindamycin | Acesulfame Potassium | Rivaroxaban | Ciclopirox ethanolamine | Daunorubicin HCl | Rupatadine Fumarate |
| | 0.82979 | 0.845618 | 0.699031 | 0.734113 | 0.695252 | 0.692771 | 0.738129 | 0.652256 | 0.353886 | 0.699386 |
| | Tioxolone | Clorsulon | Ibrutinib (PCI-32765) | Tofacitinib (CP-690550,Tasocitinib) | Epiandrosterone | Cobicistat (GS-9350) | Prostaglandin E2 (PGE2) | Rimonabant | Pravastatin sodium | Azelnidipine |
| | 0.752067 | 0.862745 | 0.589653 | 0.68344 | 0.729506 | 0.787975 | 0.716513 | 0.656863 | 0.70919 | 0.587881 |
| | Dehydroepiandrosterone (DHEA) | Arecoline HBr | Nilvadipine | Istradefylline | Apalutamide (ARN-509) | S-Ruxolitinib (INCB018424) | Paroxetine HCl | Cabazitaxel | Bepotastine Besilate | Alverine Citrate |
| | 0.750532 | 0.772502 | 0.775927 | 0.622608 | 0.720529 | 0.665958 | 0.674935 | 0.54524 | 0.69348 | 0.644815 |

| | | | | | | | | | | |
|---|---|---|---|---|---|---|---|---|---|---|
| | Idebenone | Noradrenaline bitartrate monohydrate | Dacomitinib (PF299804, PF299) | Torcetrapib | Baricitinib (LY3009104, INCB028050) | Lumiracoxib | Zaltoprofen | Bufexamac | Fosaprepitant dimeglumine salt | Azilsartan Medoxomil |
| | 0.743917 | 0.659225 | 0.747224 | 0.682377 | 0.65828 | 0.678951 | 0.679896 | 0.702103 | 0.610914 | 0.662887 |
| | Mifepristone | Fostamatinib (R788) | Niraparib (MK-4827) | Sofosbuvir (PSI-7977, GS-7977) | Carfilzomib (PR-171) | Pirfenidone | Pazopanib | Lamotrigine | Rofecoxib | Medetomidine HCl |
| | 0.657571 | 0.61115 | 0.486944 | 0.665722 | 0.46858 | 0.724427 | 0.73872 | 0.685802 | 0.720647 | 0.69407 |
| Panel 4 | Bleomycin sulfate | Chlorhexidine2HCl | Atovaquone | Pyridoxine HCl | Biotin (Vitamin B7) | Entacapone | Tylosin tartrate | Brinzolamide | Ropinirole HCl | Iopromide |
| | 0.977513 | 0.919766 | 0.961239 | 0.942865 | 0.930615 | 0.896491 | 0.939365 | 0.910841 | 1.033511 | 0.993263 |
| | Clofarabine | Piracetam | Etravirine (TMC125) | Vitamin C | Sulfamerazine | Estradiol valerate | Benztropine mesylate | Carbenicillin disodium | Ticarcillin sodium | Fexofenadine HCl |
| | 1.038411 | 0.977688 | 0.93569 | 0.966489 | 1.016887 | 0.982238 | 0.982238 | 1.078485 | 1.132033 | 1.181731 |
| | Dacarbazine | Vanillin | Ulipristal | Sulfathiazole | Sulfamethazine | Articaine HCl | Altrenogest | Eletriptan HBr | Azlocillin sodium salt | Moclobemide (Ro 111163) |
| | 1.037886 | 1.006737 | 1.058885 | 0.967889 | 1.057485 | 0.960014 | 0.960014 | 1.007962 | 1.136057 | 1.201155 |
| | Dexrazoxane HCl (ICRF-187, ADR-529) | Chlorthalidone | Indacaterol Maleate | Oxybutynin chloride | Sodium salicylate | Gliquidone | Ampicillin sodium | Flumequine | Reboxetine mesylate | Triptolide (PG490) |
| | 1.041211 | 0.952139 | 1.011287 | 1.009362 | 1.073935 | 0.987313 | 0.987313 | 0.952314 | 1.065885 | 0.833319 |
| | Epinephrine HCl | Dexmedetomidine | 2-Thiouracil | Doxepin HCl | Methylthiouracil | Butenafine HCl | Anagrelide HCl | Amitriptyline HCl | Triflusal | Borneol |
| | 1.051011 | 1.009537 | 1.028086 | 0.984338 | 0.983638 | 1.013562 | 1.013562 | 1.07096 | 1.047511 | 1.133782 |
| | Diclofenac Potassium | | Moguisteine | Ornidazole | | Mepivacaine HCl | Antipyrine | Adrenalone HCl | Trifluoperazine 2HCl | Fangchinoline |
| | 1.016537 | | 1.126958 | 0.971389 | | 1.046286 | 1.046286 | 0.988363 | 1.092134 | 0.916441 |
| | Diclofenac Diethylamine | Tazobactam | Nadifloxacin | Dexamethasone Acetate | Milnacipran HCl | Ethynodiol diacetate | Atomoxetine HCl | Azatadine dimaleate | Catharanthine | Berbamine (dihydrochloride) |
| | 1.031236 | 1.007612 | 1.110858 | 1.005337 | 0.953189 | 1.009187 | 1.009187 | 0.972264 | 1.034386 | 1.112608 |
| | Naloxone HCl | Beclomethasone dipropionate | Pidotimod | Trimethoprim | Darifenacin HBr | Sertaconazole nitrate | Betahistine 2HCl | (+,-)-Octopamine HCl | Meptazinol HCl | (+)-Fangchinoline |
| | 1.047861 | 1.016712 | 0.965614 | 1.094934 | 1.028961 | 0.971039 | 0.971039 | 0.915566 | 0.918366 | 0.791145 |
| Panel 5 | Levothyroxine sodium | Cinnamaldehyde | Echinacoside | Imperatorin | Panaxatriol | Harmine | Isopsoralen | Hederacoside C | Nonivamide | (+)-Borneol |
| | 0.903485 | 0.890477 | 0.878754 | 0.890638 | 0.83957 | 0.774209 | 0.843424 | 0.874739 | 0.864301 | 0.837803 |
| | Sodium benzoate | Tanshinone IIA sulfonate (sodium) | Notoginsenoside R1 | Scutellarin | D-Galactose | Guaiacol | Bornyl acetate | Lathyrol | Valproic acid | Vanillyl Butyl Ether |
| | 1.024731 | 0.925646 | 0.872973 | 0.867673 | 0.926289 | 1.01622 | 0.991489 | 0.960816 | 1.038381 | 1.033242 |

| | | | | | | | | | | |
|---|---|---|---|---|---|---|---|---|---|---|
| | Quinidine sulfate | Palmatine | Carvacrol | Ginsenoside Re | Glucosamine sulfate | Indigo | Sophoridine | Ginsenoside Rg1 | L-Cycloserine | Nifuratel |
| | 1.026819 | 1.008511 | 0.972539 | 0.948932 | 1.045768 | 1.008993 | 0.982496 | 0.94572 | 0.995985 | 0.981532 |
| | 4-Hydroxybenzoic acid | 5-Hydroxymethylfurfural | Succinic acid | Harmine hydrochloride | Camphor | Scopoletin | Hydroxy Camptothecine | Ginsenoside Rb1 | Mesterolone | Flavone |
| | 1.003533 | 0.931428 | 0.990204 | 0.830095 | 0.904448 | 1.011241 | 0.743376 | 0.991649 | 0.966758 | 1.014935 |
| | Betaine | Tyrosol | Palmitic acid | Quercitrin | Tetrahydropalmatine hydrochloride | Protopine | Hederagenin | (-)-Epicatechin gallate | | Histamine |
| | 1.016862 | 0.949414 | 0.905733 | 0.920829 | 1.071142 | 0.852577 | 0.991007 | 0.949414 | | 1.08158 |
| | Methyl salicylate | Ligustrazine hydrochloride | Trigonelline Hydrochloride | Loganin | Allantoin | Pyrogallol | Astragaloside IV | Forsythin | Maltitol | Veratric acid |
| | 0.798298 | 0.924683 | 0.886302 | 0.937691 | 0.904127 | 0.91296 | 0.920668 | 0.905573 | 0.955195 | 0.997912 |
| | Sinomenine hydrochloride | cis-Anethole | Stevioside | Isoquercitrin | Lawsone | L-Rhamnose monohydrate | Catalpol | Swertiamarin | Tannic acid | Vindoline |
| | 0.996467 | 0.962422 | 0.947487 | 0.96194 | 0.890959 | 0.946844 | 0.968524 | 0.952786 | 0.972378 | 1.049141 |
| | Eucalyptol | Ginkgolide C | Dehydroandrographolide | Madecassoside | Galanthamine | Arteether | α-Hederin | Liquiritin | Gamma-Oryzanol | Fusidine |
| | 0.949253 | 1.010599 | 0.923238 | 0.983299 | 0.906375 | 0.922595 | 0.925325 | 0.91023 | 0.880841 | 0.954553 |
| Panel 6 | Parecoxib | Rebeprazole sodium | Gluconolactone | Rivastigmine | Vitamin K1 | Evans Blue | Perphenazine | Sulfacetamide sodium salt hydrate | Harmaline | Daminozide |
| | 0.86234 | 0.867892 | 0.891825 | 0.879763 | 0.924182 | 0.859276 | 0.907524 | 0.871147 | 0.905418 | 0.894505 |
| | Eslicarbazepine Acetate | Sivelestat sodium tetrahydrate | Povidone iodine | Deoxycholic acid | Etretinate | Isatin | Retigabine | Cisapride hydrate | Menadiol Diacetate | Thymidine |
| | 0.952901 | 0.984875 | 1.012828 | 0.993873 | 1.049205 | 0.991767 | 1.081562 | 1.034654 | 0.986215 | 1.083668 |
| | Hydroquinidine | Lidocaine hydrochloride | Terazosin HCl | Escin | 2-Deoxy-D-glucose | Acetylcholine iodide | Retigabine 2HCl | Corticosterone | Benzyl isothiocyanate | Ceftizoxime |
| | 1.04193 | 0.955198 | 0.947731 | 0.938541 | 0.894314 | 0.916906 | 0.953858 | 0.944668 | 0.999617 | 0.999426 |
| | Glycopyrrolate | Procaine | Protirelin | Oxybenzone | Eugenol | (+)-Catechin | Salvianolic acid B | Betulin | N-Acetylneuraminic acid | Cefuroxime axetil |
| | 1.01972 | 0.936052 | 0.913651 | 0.870572 | 0.902738 | 0.919012 | 0.989853 | 0.974153 | 1.003063 | 1.004212 |
| | Tiagabine hydrochloride | Benzocaine hydrochloride | Loxoprofen | Guanfacine Hydrochloride | Oleic Acid | (-)Epicatechin | Trapidil | Dihydrotestosterone(DHT) | Drostanolone Propionate | L-Cysteine HCl |
| | 1.011105 | 0.860234 | 0.887612 | 0.850469 | 0.886655 | 0.935669 | 0.838981 | 0.931457 | 0.948114 | 0.942179 |
| | Atazanavir | Etonogestrel | Sildenafil Mesylate | D panthenol | Latanoprost | Benzenesulfonamide | Psoralen | p-Coumaric Acid | Trenbolone acetate | Diatrizoate sodium |
| | 0.954241 | 0.889336 | 0.876125 | 0.869998 | 0.863105 | 0.942179 | 0.845491 | 0.842811 | 0.943136 | 0.849129 |

| | | | | | | | | | | |
|---|---|---|---|---|---|---|---|---|---|---|
| | Fusidate Sodium | Hydroxyprogesterone caproate | Efavirenz | CarbinoxaMine Maleate | Esculetin | Lauric Acid | Ondansetron Hydrochloride Dihydrate | Melibiose | Methandrostenolone | Atenolol |
| | 0.954815 | 0.852192 | 0.847406 | 0.884166 | 0.891442 | 0.852001 | 0.791116 | 0.859659 | 0.866169 | 0.835535 |
| | Molsidomine | Tiagabine | Vitamin E | Saxagliptin hydrate | (-)-Menthol | Cinnarizine | Citalopram HBr | L-5-Hydroxytryptophan | Nicergoline | Saccharin |
| | 0.940839 | 0.856787 | 0.896611 | 0.851426 | 0.820601 | 0.803753 | 0.820793 | 0.834961 | 0.77063 | 0.80337 |
| Panel 7 | Diastase | Ibudilast | Faropenem Sodium | Iproniazid | Sulfamethoxazole sodium | Cefathiamidine | Cytosine | Maltol | Iminostilbene | Tiamulin fumarate |
| | 1.056098 | 1.034846 | 1.02757 | 1.062033 | 1.037718 | 0.971089 | 1.029868 | 0.944093 | 1.154509 | 1.911162 |
| | Maltose | Acotiamide hydrochloride | Dalbavancin | TriacetonaMine | Cefodizime Sodium | Calcium Dobesilate | Elagolix Sodium | Nonanoic acid | Fimasartan | Valpromide |
| | 1.151828 | 1.192035 | 1.152211 | 1.115834 | 1.103389 | 1.042121 | 0.943902 | 0.951943 | 1.153169 | 1.574957 |
| | Piperonyl butoxide | Mosapride | Levocetirizine Dihydrochloride | Indole-3-carboxylic acid | Pyridoxal 5-phosphate monohydrate | Lynestrenol | Sulfogaiacol | Fumaric acid | Sulfalene(SMPZ) | Methylcobalamin |
| | 1.212522 | 1.191461 | 1.126173 | 1.117748 | 1.100517 | 1.091518 | 0.962474 | 1.104155 | 1.22152 | 1.453571 |
| | Tolmetin | Laurocapram | Flucloxacillin sodium | Squalene | Cefazedone | Taurolidine | Propiverine hydrochloride | Usnic acid | Efonidipine | Tavaborole (AN-2690) |
| | 1.293509 | 1.150297 | 1.062799 | 1.143787 | 1.088072 | 1.046334 | 1.084243 | 1.016083 | 0.897186 | 1.495884 |
| | Cefoxitin sodium | Potassium acetate | Tafluprost | Cefetamet pivoxil hydrochloride | Cephapirin Benzathine | Menbutone | Proxyphylline | Linalool | Azathramycin | Avermectin B1(Abamectin) |
| | 1.325866 | 1.198736 | 1.084626 | 1.15853 | 1.076776 | 1.042121 | 1.001149 | 1.010913 | 0.973578 | 1.503925 |
| | Propantheline bromide | Cefcapene Pivoxil Hydrochloride | Gadopentetate Dimeglumine | Nicarbazin | Robenidine Hydrochoride | Nikethamide | | Glycocholic acid | Anamorelin | Tofacitinib (CP-690550) Citrate |
| | 1.239709 | 1.242964 | 1.132108 | 1.188589 | 1.088263 | 1.036186 | | 1.070649 | 1.021444 | 1.585296 |
| | Aceclofenac | Rabeprazole | Ecabet sodium | Propacetamol hydrochloride | Eperisone hydrochloride | Perospirone hydrochloride | Asunaprevir | Lactobionic acid | Sorbic acid | Fingolimod (FTY720) HCl |
| | 1.260195 | 1.259621 | 1.24507 | 1.200268 | 1.190695 | 1.143021 | 1.215776 | 1.09037 | 0.926479 | 1.54126 |
| | Nilutamide | Meropenem Trihydrate | Bedaquiline fumarate | Xanthinol Nicotinate | Neticonazole Hydrochloride | Bifendate | cis-Aconitic acid | Buparvaquone | | Tacrolimus (FK506) |
| | 1.175761 | 1.229562 | 1.200268 | 1.204289 | 1.171549 | 1.143787 | 1.166379 | 1.043079 | | 1.760674 |
| Panel 8 | Pimecrolimus | Ioversol | Efinaconazole | Phenazine methosulfate | Thiocolchicoside | Daclatasvir Digydrochloride | | Ceforanide | Febantel | Lanolin |
| | 0.98265 | 0.990458 | 0.968553 | 1.114943 | 1.047929 | 1.017133 | | 1.071134 | 1.067231 | 1.128172 |
| | Cefotiam hydrochloride | Crisaborole (AN2728) | Mebeverine Hydrochloride | Valethamate Bromide | Granisetron | Trelagliptin succinate | Donepezil | Vitamin K2 | Rafoxanide | Tylosin |
| | 1.103665 | 1.044893 | 1.040772 | 1.037736 | 1.061375 | 1.068966 | 1.088701 | 1.094123 | 1.119931 | 1.249404 |

| | | | | | | | | | | |
|---|---|---|---|---|---|---|---|---|---|---|
| | Teprenone | Simeprevir | 4-Aminopyridine | Actarit | Rifamycin sodium salt | Ganciclovir sodium | Argatroban Monohydrate | Lentinan | SulfadiMe thoxine sodium | Ademetionine |
| | 1.126654 | 1.018868 | 1.016699 | 0.974843 | 0.970722 | 0.982433 | 1.023205 | 1.044025 | 1.059206 | 1.165691 |
| | Delamanid | Isoprinosi ne | Etofylline | Tiamulin | Milbemycin Oxime | Pramipexol e dihydrochlo ride | Acotiamide | Carbazochr ome | | alpha-Arbutin |
| | 1.595532 | 0.996964 | 0.98959 | 1.006289 | 0.945348 | 0.979614 | 1.023422 | 0.996313 | | 1.072435 |
| | Brivudine | Oxyclozan ide | Dihydralazine sulphate | Difloxacin hydrochloride | Dinoprost tromethamine | Losartan | Xipamide | Azamethiph os | Pralidoxi me Iodide | Propyl gallate |
| | 1.32661 | 0.97224 | 1.039254 | 0.973542 | 0.947517 | 1.054435 | 1.045977 | 0.83496 | 0.974409 | 1.00694 |
| | Indometacin Sodium | Indobufen | Mephenesin | Bevantolol hydrochloride | Revaprazan Hydrochloride | Dabrafenib Mesylate | Regorafenib Monohydrate | p-Anisaldehy de | Stachyos e | Hydroquinine |
| | 1.096725 | 0.992626 | 0.951204 | 0.9293 | 0.96118 | 0.934721 | 0.9293 | 0.887877 | 0.934721 | 1.035784 |
| | | Tilorone dihydrochl oride | Terconazole | Benorylate | Pixantrone Maleate | Mupirocin calcium | Osimertinib mesylate | Tianeptine | lutein | Doramectin |
| | | 0.743656 | 0.926046 | 0.925613 | 0.664064 | 0.905227 | 0.774886 | 0.845153 | 0.873563 | 0.923661 |
| | Octenidine Dihydrochloride | Nadolol | Melitracen hydrochloride | Clonixin | Metadoxine | Duloxetine | Sitagliptin | Geranyl acetate | Proanthoc yanidins | Olivetol |
| | 0.78638 | 0.804381 | 0.85231 | 0.855129 | 0.883322 | 0.911299 | 0.888094 | 0.914986 | 0.888961 | 0.980265 |
| Panel 9 | Nitisinone | Nerolidol | Butoconazole | Ethoxyquin | Betrixaban maleate | Dasabuvir( ABT-333) | Sultamicillin | Carbaryl | Hyperosid e | Dantrolene sodium |
| | 0.768805 | 0.730349 | 0.814644 | 0.788956 | 0.818028 | 0.740809 | 0.748346 | 0.756191 | 0.768651 | 0.659591 |
| | Dolasetron | Cefpodoxi me proxetil | Diflorasone | Ajmaline | Mepivacaine | Ombitasvir (ABT-267) | Ertugliflozin | Promazine hydrochlori de | Saikosap onin D | Cloperastine hydrochloride |
| | 0.865405 | 0.791571 | 0.87002 | 0.985387 | 0.821104 | 0.926319 | 0.976004 | 0.931088 | 0.608676 | 0.767267 |
| | Meisoindigo | Cefmetaz ole sodium | Bendazac | Methyl Aminolevulinat e Hydrochloride | Cyclofenil | Paritaprevir (ABT-450) | Diflucortolone valerate | Metoprolol | Curculigo side | Clidinium Bromide |
| | 0.819874 | 0.942932 | 1.009076 | 1.06199 | 0.862175 | 0.993232 | 0.947085 | 0.973543 | 0.776342 | 0.81249 |
| | Gamithromycin | Cefminox Sodium | Pikamilone | Dibutyl phthalate | Phenolphthale in | Propylpara ben | | Quinacrine Dihydrochlo ride Dihydrate | Aucubin | Molindone hydrochloride |
| | 0.965236 | 0.978926 | 1.013844 | 1.018766 | 0.934779 | 0.849408 | | 0.598369 | 1.025996 | 0.854945 |
| | Ceftezole sodium | Cefpirami de sodium | Alogliptin | Dimethyl phthalate | Chlorhexidine | Sultamicilli n Tosylate | Metyrapone | Berberine Sulfate | Saikosap onin A | Prilocaine hydrochloride |
| | 0.987233 | 0.957699 | 0.990771 | 1.075681 | 0.740963 | 0.864636 | 0.991694 | 0.761575 | 0.832795 | 0.943701 |
| | Sulbenicillin Sodium | Ceftiofur | Fipronil | Formate | Nefazodone hydrochloride | Squalane | Parecoxib Sodium | Triprolidine Hydrochlori de | Pivmecilli nam hydrochlo ride | Tribenzagan Hydrochloride |
| | 0.950315 | 0.979695 | 0.978465 | 0.973696 | 0.93401 | 0.963698 | 0.931088 | 1.008306 | 1.027073 | 0.904784 |

| | | | | | | | | | | |
|---|---|---|---|---|---|---|---|---|---|---|
| | Metroprolol succinate | Safinamide | Ethyl Oleate | Imidafenacin | Chlorprothixene hydrochloride | Isoprene | 1,4-Cineole | Sofalcone | Rolapitant | Rimantadine Hydrochloride |
| | 0.970158 | 0.954315 | 1.002154 | 1.01892 | 0.916013 | 0.983849 | 0.956314 | 0.954776 | 0.794186 | 0.857868 |
| | Vanillic acid | Regadenoson | Lactitol | Betrixaban | Tegaserod Maleate | Chloramphenicol sodium succinate | Clindamycin alcoholate | Sanguinarine chloride | Gefarnate | Desipramine Hydrochloride |
| | 0.950777 | 0.966928 | 0.985848 | 0.956314 | 0.503615 | 0.982926 | 0.941701 | 0.510537 | 0.91186 | 0.82818 |
| Panel 10 | Fluorometholone | Iopanoic acid | Trimebutine maleate | Erythromycin estolate | 1, 10-Phenanthroline monohydrate | Pamabrom | Xylazine | Olmesartan | (1R)-(-)-Menthyl acetate | Bedaquiline |
| | 0.626848 | 0.868683 | 0.810072 | 0.745789 | 0.691818 | 0.811103 | 0.753867 | 0.788759 | 0.790822 | 0.838432 |
| | Cefoperazone sodium | Betahistine mesylate | Dehydroepiandrosterone acetate | Cinchocaine | D-Ribose | (-)-Sparteine Sulfate | Triflupromazine hydrochloride | Cytarabine hydrochloride | p-Cymene | Ammonium lactate |
| | 0.664146 | 1.045892 | 1.040048 | 1.059986 | 0.990718 | 1.067721 | 0.920935 | 1.001375 | 0.883293 | 0.877793 |
| | Fluorescein | Amodiaquine hydrochloride | 4-Aminophenol | Moxifloxacin | Sulfacetamide | D-Pantothenate Sodium | Dapagliflozin propanediol monohydrate | Cinnamyl acetate | Sodium cholate | Benzalkonium chloride |
| | 0.854417 | 0.93692 | 0.972843 | 0.999141 | 1.007906 | 1.032657 | 0.964936 | 0.953592 | 0.943795 | 0.739945 |
| | Disopyramide | Hydrocortisone acetate | Penicillin G Procaine | Tizanidine | Hydroxylammonium chloride | Tetrahydropalmatine | Trimethadione | Citronellal | Diphenylamine Hydrochloride | Amsacrine hydrochloride |
| | 0.896184 | 1.017704 | 1.139395 | 1.009625 | 0.943795 | 0.96614 | 0.996219 | 0.955827 | 1.033689 | 0.872637 |
| | Lomefloxacin | Ilaprazole sodium | Salmeterol | Tropisetron | Ethyl gallate | Midecamycin | Tocofersolan | Camphene | (+)-Longifolene | Cefotiam Hexetil Hydrochloride |
| | 0.901684 | 1.006531 | 1.026126 | 0.982812 | 0.992953 | 1.055861 | 0.923169 | 0.960639 | 1.023032 | 1.064455 |
| | Econazole | Ropivacaine | Acetophenone | Olprinone | Amenamevir | Ethacrynic Acid | Anisole | Vitamin A | Hippuric acid | Cefozopran hydrochloride |
| | 0.977484 | 0.943623 | 0.94603 | 1.024063 | 0.95978 | 0.937264 | 0.987796 | 0.985046 | 0.981265 | 0.927466 |
| | Atropine sulfate | 2'-deoxyuridine | Geraniol | Landiolol hydrochloride | Kasugamycin hydrochloride | 2-Hydroxybenzyl alcohol | Tilmicosin phosphate | α-Terpineol | Betahistine | |
| | 0.944826 | 0.80801 | 0.952217 | 0.911138 | 0.873668 | 0.79254 | 0.882606 | 0.838604 | 0.868512 | |
| | Salbutamol | Vortioxetine | Doripenem | Dimetridazole | Lanatoside C | Thioridazine hydrochloride | Arabic gum | (1S)-(-)-α-Pinene | Cilastatin | Emedastine Difumarate |
| | 0.902544 | 0.637161 | 0.794775 | 0.79254 | 0.51461 | 0.717944 | 0.719663 | 0.718288 | 0.790993 | 0.814025 |
| Panel 11 | Iguratimod | Diphenylpyraline hydrochloride | Drofenine Hydrochloride | Methyl linolenate | Phthalylsulfathiazole | Pravastatin | Oxantel Pamoate | Stearic acid | Deferoxamine mesylate | Citric acid |

| | | | | | | | | | |
|---|---|---|---|---|---|---|---|---|---|
| 0.978947 | 0.975329 | 0.967763 | 0.983224 | 0.974178 | 0.932895 | 0.943421 | 0.950658 | 0.941941 | 0.917763 |
| Hydroxyzine pamoate | Ertapenem sodium | Moxisylyte hydrochloride | Isoproterenol sulfate dihydrate | Alvimopan dihydrate | lurasidone | Dichlorophene | Midodrine hydrochloride | Morantel tartrate | Methyl Oleate |
| 1.044243 | 0.984539 | 0.949507 | 0.975 | 0.978783 | 1.01102 | 1.010526 | 0.978059 | 1.062007 | 0.94227 |
| | Omeprazole Sodium | Fruquintinib | Desoximetasone | Fenipentol | atorvastatin | Triiodothyronine | Midodrine | Chlorpromazine | D-Mannose |
| | 0.954276 | 0.957895 | 0.972204 | 0.966612 | 0.973191 | 0.916941 | 0.939309 | 1.004934 | 1.233553 |
| Dexrazoxane | Emedastine | Isoxsuprine hydrochloride | Tropic acid | Carvedilol Phosphate | Abemaciclib | Tetryzoline | Benzathine penicilline | Dihydroergotamine Mesylate | Sodium Dehydrocholate |
| 1.029112 | 0.969901 | 0.95 | 0.94227 | 1.010691 | 0.834046 | 0.973026 | 0.997697 | 0.966283 | 0.950987 |
| Elbasvir | Tiaprofenic acid | Chloropyramine hydrochloride | Mexenone | Raceanisodamine | Acetohexamide | Delapril Hydrochloride | Diclofenac Epolamine | Baricitinib phosphate | Alfuzosin |
| 1.052632 | 0.990954 | 1.009046 | 1.024671 | 1.00477 | 1.049342 | 1.000822 | 0.998684 | 1.073026 | 1.412829 |
| Indigo carmine | Ranitidine | Mivacurium chloride | Levomilnacipran Hydrochloride | Norgestrel | Acrivastine | Fosfosal | Nebivolol | Methyl Stearate | Aliskiren |
| 1.028947 | 0.99227 | 1.006414 | 1.014309 | 1.016612 | 1.052961 | 1.042599 | 1.026809 | 0.985855 | 1.028125 |
| Indacaterol | Minaprine dihydrochloride | Dolasetron Mesylate | Isopropamide Iodide | Ambroxol | Ceftizoxime sodium | Alimemazine Tartrate | Palonosetron | Isoeugenol | Fenoterol hydrobromide |
| 1.019737 | 1.006086 | 0.948684 | 0.959539 | 0.997204 | 0.957072 | 0.993421 | 0.935197 | 0.967599 | 0.960691 |
| Venlafaxine | Orphenadrine Hydrochloride | (-)-Verbenone | Ketorolac tromethamine salt | | Glecaprevir | Sebacic acid | Quetiapine | Methyl linoleate | Fenoterol |
| 1.039803 | 0.952632 | 0.990625 | 0.99852 | | 1.061842 | 1.008388 | 0.904112 | 0.989967 | 0.974836 |
